# Rethinking Intelligence: Implications for Teachers and Students in Barbadian Schools

**DOI:** 10.3390/jintelligence13090121

**Published:** 2025-09-19

**Authors:** Jason Marshall, Garry Hornby

**Affiliations:** 1 School of Education, Faculty of Humanities and Education, The University of the West Indies, Cave Hill Campus, Bridgetown P.O. Box 64, Barbados; 2 Institute of Education, University of Plymouth, Drake Circus, Plymouth PL4 8AA, UK; garry.hornby@plymouth.ac.uk

**Keywords:** intelligence theory, high-stakes examinations, intelligence testing, education reform

## Abstract

In most Western societies, intelligence testing has evolved beyond simple measures of language and numerical abilities. Although these measures are valuable in predicting academic achievement and career success, it is widely recognized that modern intelligence assessments offer a more comprehensive view of intellectual aptitude. Unfortunately, in Caribbean Small Island Developing States, like Barbados, despite ongoing efforts towards educational reform and an increasing body of research and related theories advocating for inclusive approaches to understanding and nurturing students’ intellectual development, the education system remains heavily influenced by traditional conceptualizations of intelligence that present a somewhat narrow view of students’ aptitudes. This perspective appears to consider students’ performance on high-stakes examinations measuring numerical and language abilities as perhaps the most indicative markers of intelligence. Building on the work of renowned educational theorists such as Sternberg, Renzulli, and Gardner, and drawing from literature on traditional and contemporary measures of intelligence, this opinion paper examines the implications of deconstructing and redefining traditional views of intelligence within the Barbadian educational context. The value of conventional measures and the potential challenges and limitations associated with transitioning to contemporary intelligence assessments are acknowledged, and the pedagogical and assessment implications at primary and secondary school levels are discussed.

## 1. Introduction

It has been suggested that there is a silent crisis in the functioning of educational systems across the Caribbean (De Lisle 2012; Hornby 2020; Warrican 2021). The traditional view of intelligence, which disproportionately emphasizes cognitive and academic abilities, continues to significantly influence the structure and implementation of school-based education and assessment practices in the region. This has led to the establishment and entrenchment of educational systems that perpetuate social and economic inequalities that are deeply rooted in the region’s colonial history (Hornby 2023; Warrican 2021). In Caribbean nations, such as Barbados, where enduring economic and educational disparities are evident, it is important to examine how conventional and historical perspectives of intelligence continue to influence the island’s educational system and student outcomes. An understanding of these dynamics necessitates contextualizing the discourse on intelligence within a broader historical, regional, and global framework.

Intelligence is a pervasive and multidisciplinary concept. Although it is most prominently featured in fields such as psychology and education, it also permeates several other disciplines. Thus, an individual considered successful in a particular endeavour (e.g., science, politics, or technology) is usually considered intelligent. Furthermore, research illustrates the value of intelligence in predicting individual success (Squalli and Wilson 2014). However, despite the pervasiveness of the concept of intelligence, there is little consensus about its elements or key functions. For example, “some see intelligence as biologically based, whereas others view it as a cultural invention, and how a given culture chooses to define intelligence. Some see intelligence as basically a unitary phenomenon, whereas others see it as a multiple phenomenon” (Sternberg 2019, p. 1).

During the early 20th century, intelligence emerged as one of psychology’s most groundbreaking, controversial, and heavily researched constructs. Nonetheless, it was celebrated for its ability to help identify individual differences in cognitive ability. The early forerunners in the intelligence movement focused on the cognitive processes involved in intelligence. They identified reasoning, judgment, and abstract thought as critical aspects of an individual’s intellectual aptitude. For example, Binet and Simon identified the components of intelligence as reasoning, judgment, memory, and the power of abstraction.

In 1904, Spearman developed the Two-Factor Theory of Intelligence. He suggested that intelligence comprises a ‘g’ factor (general intelligence) and an ‘s’ factor (specific intelligence). He proposed that achievement on various intellectual tasks (e.g., math, English) is influenced by an underlying ‘g’ factor and a specific ‘s’ ability related to the task in question. People with a strong ‘g’ factor were considered likely to perform well on various cognitive tasks. Although other theories (e.g., Thurstone’s Theory of Primary Mental Abilities and Cattell’s Theory of Crystallized and Fluid Intelligence) emerged that either disputed or supported Spearman’s research findings, it was generally agreed that the degree of someone’s intelligence was determined by how well they performed on various cognitive tasks.

Pioneering research on intelligence in the 20th century significantly enhanced human understanding of mental ability; however, it also had several drawbacks. For example, Ayres (1911) questioned Binet’s intelligence test based on overemphasizing abstraction rather than emphasizing practical ability. Contemporary intelligence theorists, such as Sternberg (1985) and Gardner (1987), have critiqued early views of intelligence for their heavy focus on mathematical and reasoning abilities, while overlooking other forms of intelligence (e.g., practical, musical, emotional).

Despite significant variations, disagreements, and criticisms about early and traditional understandings of intelligence, conventional perspectives and practices related to intelligence remain prominent in the English-speaking Caribbean education systems, especially in Small Island Developing States, such as Barbados. This traditional view of intelligence continues to shape the island’s educational context and practices, leading to significant implications for teaching and learning. However, many of these educational practices are also rooted in the country’s colonial history, which was driven and shaped by the philosophies of segregation and social conditioning.

## 2. The History of Education in Barbados

To comprehend the reasons underpinning the enduring prominence of traditional views of intelligence within the Barbadian educational context, it is imperative to acknowledge that educational practices on this island are inextricably linked to the nation’s colonial legacy. The prevailing educational system encapsulates the historical legacies of colonialism, which aimed to prevent the access of Black children to education via the implementation of restrictive policies and practices (Brissett 2022; De Lisle 2012). This historical context resulted in an elitist educational framework that sought to differentiate students, not solely based on their race, but also according to their academic capabilities. Although overt barriers to education have been significantly dismantled, this historical backdrop continues to exert influence over critical aspects of Barbadian education, which carry significant implications for student success and perceptions of intellectual capacity.

Formal education in Barbados dates back to 1686, when the first school was established for poor white children, while wealthy planters sent their children to England to receive an education. It was not until the post-emancipation era, after the year 1838, that educational opportunities for children of African descent were contemplated. Nevertheless, such education was predominantly restricted to the three R’s and was intricately linked to moral and religious instruction (Warrican et al. 2024). This approach was perceived as a mechanism for civilizing individuals of African descent and sustaining British dominance.

During the twentieth century, access to education in Barbados expanded. The demand for and expansion of education on the island also brought issues related to the implementation of school placement systems that were inherently designed to separate and identify students with varying levels of intelligence. Additionally, they also reinforced economic and social hierarchies (Pilgrim et al. 2018). Before 1959, each secondary school in Barbados had its own entrance examination and there were limits to the number of students who could transition to secondary school. It was considered that a ‘fairer’ system of allocation was required.

Therefore, in 1959, the Ministry of Education introduced the Common Entrance Examination to facilitate the transition of all students from primary to secondary school between the ages of 10 and 12. It has been over six decades since the introduction of this examination. Despite persistent calls for its abolishment due to perceived inequities and the perpetuation of social inequalities, it continues to be used for secondary transfer. Students are assessed in two subject areas—Mathematics and English, and unfortunately, student performance on this exam is considered to be indicative of their intelligence levels.

## 3. High-Stakes Examinations in Barbados and Traditional Views of Intelligence

The history of education in Barbados has led to the development of an education system driven by traditional conceptualizations of intelligence. In this system, students’ intellectual aptitudes are considered to be determined by their performance on high-stakes examinations, such as the Common Entrance Examination (CEE), taken around the age of 11, and the Caribbean Secondary Education Certificate (CSEC), taken around the age of 16. Students who obtain higher scores on the CEE transition to older, more prestigious secondary schools on the island and are perceived to be academically inclined and more intelligent than their counterparts with lower scores who attend the newer secondary schools (Warrican et al. 2024).

The emphasis on academic ability as a gauge of intelligence is what Sternberg (2021) refers to as general intelligence. He describes it as a type of intelligence that emphasizes cognitive ability. “It includes cognitive skills such as vocabulary acquisition and knowledge, inductive reasoning, spatial visualization, memory, and perceptual speed” (Sternberg 2019, p. 2). Sternberg argues that this type of intelligence is typically assessed by cognitive tests that are decontextualized from everyday realities, driven by the desire to attain high scores, and are time-bound. His description of general intelligence reflects the selective secondary transfer of the education system and conventional high-stakes assessment practices in Barbados.

Evidence of this is seen in the CEE, where heavy emphasis is placed on assessing students’ linguistic and mathematical abilities. This narrow view of assessing academic ability and thereby indicating intelligence was questioned and continues to be challenged by renowned scholars. For example, Gardner and Hatch (1989), “were disturbed by the nearly exclusive stress in school on two forms of symbol use: linguistic and logical-mathematical. They argued that although these two forms are important in a scholastic setting, other varieties of symbol use also figure prominently in human cognitive activity within and especially outside of school” (p. 5).

Unfortunately, assessments on the CEE still focus exclusively on students’ linguistic and mathematical abilities, and the scores on this examination are considered indicative of students’ intellectual prowess. This view is reported by Warrican et al. (2024) who note that high scores on this examination are considered to indicate students with high intelligence capable of benefiting from a more academic school-based education, while low scores suggest a lack of sufficient intellectual power to benefit from this type of provision, hence relegating these children to less academically focused secondary schools.

Students who do well on the CEE are highly celebrated in Barbados. The best-performing boys and girls, as well as the top-performing schools, are highlighted in the media each year for achieving the highest scores, dominating the news cycle during the examination period. Their performance is viewed as a clear indication of their superior cognitive ability and a welcome indicator of their life trajectory. Thus, the promise and hope of future life success are deeply intertwined with the myopic view that the CEE provides an accurate gauge of a child’s intelligence.

However, many prominent Caribbean educators have challenged the notion that test-taking, which emphasizes mathematical and linguistic abilities, is the most effective gauge of intelligence. De Lisle (2012), for example, argues that, “the persistence of test-based early selection in Barbados points to a widespread and implicit belief in the infallibility of test scores” (p. 109). Warrican (2021) shares a similarly critical view. He argues that this approach to assessment maintains a strong bias in favour of students from middle and upper-class backgrounds. Warrican’s position parallels earlier concerns about intelligence testing, where the lack of cultural sensitivity in the design of intelligence tests has been viewed as a major flaw in their validity and a significant contributor to inequality and injustice (Holden and Tanenbaum 2023).

Although concern remains about the CEE’s inequities and the declining academic performance of students in the CSEC at the secondary level, there is evidence to suggest some recognition that, over the years, academic ability has been overemphasized as a primary measure of a student’s intellect. The country’s renewed push for education reform and the clarion call for implementing alternative pathways to transition students to secondary school are evidence of this acknowledgement (Pilgrim et al. 2018).

The introduction of the Caribbean Vocational Qualification (CVQ) into some secondary schools in Barbados serves as evidence of an effort to rethink how academic achievement is viewed. Notwithstanding the aim of this initiative, which was to introduce a competency-based framework to secondary education, designed to enhance students’ technical and vocational skills, while providing opportunities for certification, it also challenges traditional notions of intelligence. For example, the emphasis on acquiring practical skills aligns with Gardner’s bodily kinesthetic intelligence and Sternberg’s practical intelligence. While educational innovations, such as the CVQ, point to future improvements, systemic issues rooted in the education system continue to stifle progress. One such issue is the mismatch between the firmly entrenched views of traditional intelligence and the essentials of 21st-century learning.

It is widely acknowledged that intelligence in the 21st century can no longer be assessed merely by a student’s performance on high-stakes examinations, particularly those that predominantly emphasize mathematical and linguistic abilities. The demands of the 21st-century knowledge economy necessitate that students are equipped with skills such as problem-solving, innovation, critical thinking, effective communication, collaboration, and digital literacy. Many of these skills are not assessed by traditional measures of intelligence.

To aid students in attaining the required 21st-century skills, it is imperative to implement educational reform that prioritizes the design of assessments and the creation of learning environments that act as catalysts in facilitating the development of these skills. This viewpoint is grounded in the Framework for 21st Century Learning (Battelle for Kids 2019), which underscores the essential skills and support systems required within this new knowledge economy. The framework emphasizes the importance of key 21st-century skills, including learning and innovation skills, information, media, and technology skills, as well as life and career skills. Furthermore, the framework stipulates that for these skills to be effectively cultivated, robust support systems must be in place, which include 21st-century standards, curriculum, and instruction tailored for the 21st century, professional development for educators, and modern learning environments. While the framework identifies the three Rs as a vital component of 21st-century learning, it advocates that the curriculum must transcend this foundational focus.

Most notably, primary education in Barbados remains focused on the 3Rs (reading, writing, and arithmetic). This emphasis, while important, also compromises opportunities for students to display other skills and potential they may have and encourages teachers to focus on teaching to the test, which limits students’ exposure to other critical 21st-century skills. This view is also expressed by Pilgrim et al. (2018), who note that, “a key issue is the narrowing of the curriculum, and the use of teacher-centered rather that student-centered pedagogy… such a focus leads to the neglect of important curriculum areas not prioritized by the CEE, for example, higher order thinking skills and other critical skills… the ability to work as a part of a team to co-create knowledge in online as well as face-to-face contexts” (p. 116).

A crucial aspect in shaping a teacher’s teaching style is the standard they consider the ‘best indicator’ of a student’s abilities. In Barbados, it appears that this standard is determined by student performance in summative assessments, which are often used to gauge a student’s academic skill and, consequently, their intellectual potential. Thus, a teacher’s understanding of intelligence plays a key role in the learning environments they create.

However, while many scholars (see, for example, Flynn 1987; Gardner 1987; Sternberg 2021) have criticized traditional views and measures of intelligence, and more contemporary measures (e.g., Weschler’s Intelligence Test and the Kauffman Brief Intelligence Test) have emerged over the decades, several empirical studies have highlighted the predictive value of traditional intelligence tests (Gygi et al. 2017; Kato and Scherbaum 2023).

Although the concerns highlighted above emphasize critical issues that need to be addressed to improve education in Barbados, a one-dimensional approach cannot be adopted. Therefore, policy makers and education practitioners, in moving toward change, must be careful not to overlook the inherent value of traditional measures of intelligence and ability testing in predicting academic and personal success.

### The Value of Traditional Views and Measures of Intelligence

Despite the inherent limitations of traditional views and measures of intelligence that overemphasize mathematical and linguistic abilities, recent research (see, for example, Gygi et al. 2017; Breit et al. 2024; Kato and Scherbaum 2023) demonstrates that there is still value in the predictive power of such measures that cannot be overlooked or underestimated. For example, research by Berkowitz and Stern (2018) found that students’ mathematics and verbal reasoning abilities were good predictors of their performance in STEM subjects. Similarly, recent research by Breit et al. (2024) indicates that strong reasoning and mathematical ability, as measured by intelligence tests, are statistically significant predictors of students’ performance in mathematics and other subject areas. Furthermore, a study by Kato and Scherbaum (2023) on cognitive ability found that the measures of quantitative and verbal abilities were good predictors of job performance.

The above literature highlights the value of traditional intelligence measures in providing a foundation for understanding and predicting students’ scholastic aptitude and potential for career success. As such, any consideration of dismissing the inherent benefits of similar ability testing measures because of their limited scope (i.e., the focus on mathematical and linguistic abilities) must be approached with caution.

This view is supported by recent data emerging from a study by Friedman et al. (2024). The findings suggest that high-stakes tests such as the Scholastic Assessment Test (SAT) and the American College Testing (ACT) are good predictors of college students’ GPA. Perhaps, therefore, rather than undermining the value of these measures, the focus should be on how, through effective teaching, to get students to reach the standards required to excel inside and outside of school.

Furthermore, countries like Singapore, renowned for their high-achieving educational systems, continue to utilize high-stakes examinations such as the Primary School Leaving Examination (PSLE), which emphasize fundamental aspects related to intelligence, including numeracy and language skills, to assess students’ academic capabilities and potential for future academic success (Loh and Shih 2016). However, this practice has not been exempt from criticism. Tan (2025), for example, argues that the system perpetuates school-based segregation and other forms of inequity. Regardless of the variations in scholastic opinions about intelligence and ability testing, how teachers view intelligence has important implications for the teaching and learning process (Gullo et al. 2025; Robinson and Bond 2025).

## 4. Implications of Traditional Views and Measures of Intelligence for Teaching and Learning in the Barbadian Context

Teachers’ perceptions of intelligence have significant implications for their teaching practices. For example, Shearer (2018) posits that teachers who subscribe to the IQ tradition, which argues that intelligence is primarily associated with academic skills (e.g., reading, math, and writing) are more likely to engage in teacher-centered instruction, such as ‘teaching to the test’. Contrastingly, he argues that, “teachers who believe that human intelligence cannot be summed up with a single number, recognize that it involves more than scholastic aptitude and seek to increase student learning through differentiated instruction that emphasizes strength-based activities” (p. 1). This often results in these teachers creating more inclusive learning environments.

Furthermore, recent research (Gullo et al. 2025; Robinson and Bond 2025) on teachers’ perceptions of intelligence has identified a form of Pygmalion effect. Specifically, educators who perceive intelligence as innate and immutably fixed tend to be less inclined to modify their pedagogical approaches and tend to establish classroom environments that do not promote student agency. Such educators often depend on performance-centered instructional methods (Laine and Tirri 2023).

In Barbados, there is a strong focus on scholastic ability as a measure of intelligence. Teachers often lament that it is challenging to deliver more inclusive forms of instruction due to the emphasis on covering content that prepares students for high-stakes examinations. Teachers frequently express difficulties in implementing growth-mindset pedagogies, due to heavy syllabi and an emphasis on preparing students for high-stakes examinations (Leacock 2020). The consequence of this approach is exclusive teaching practices. These practices lead to a teacher-centered, exam-focused pedagogy that prioritizes a select few students over the broader student body. This is a clear reflection of exclusive and elitist instruction rooted in colonial-era systems (De Lisle 2012; Hornby 2023; Pilgrim et al. 2018; Warrican 2021). Thus, students who are less academically able are left behind and seen as generally less capable of learning. Such experiences can harm their academic self-concept and academic self-efficacy, ultimately undermining their motivation to learn (Wang and Yu 2023).

## 5. Contemporary Views and Measures of Intelligence

In the mid-to-late 20th century, contemporary theories and measures of intelligence emerged that challenged perspectives based on IQ measurements. Esteemed scholars (e.g., Gardner 1987; Renzulli 1988; Sternberg 1985) in the field of intelligence have articulated significant criticisms of traditional notions of intelligence due to their substantial emphasis on academic capabilities while overlooking other forms of intelligence. For example, the work of Renzulli, although focused on giftedness, also aimed to reconceptualize conventional views of intelligence by highlighting that intelligence is an integral part of giftedness, while it is not all that is required to be gifted. While giftedness is often associated with intelligence and academic ability, it is essential to recognize that not all gifted students perform optimally within the educational system (Van Hooijdonk et al. 2025). Nonetheless, if they receive appropriate educational conditions, such as differentiated instruction or a compacted curriculum, they can excel (Renzulli 2016). Therefore, educators should reconsider and question conventional views of intelligence and giftedness that may limit the range of teaching strategies used in the classroom.

In Barbados, for example, educators’ perceptions of giftedness and intelligence seem to be limited to what Renzulli (2016) refers to as ‘schoolhouse giftedness’. This type of giftedness emphasizes cognitive ability and performance on standardized examinations. Students who fall into this category of giftedness typically excel on traditional school examinations, such as the CEE and CSEC. However, Renzulli suggests that we should be cautious in concluding that this form of giftedness is the only type that will lead to school success. Instead, he proposes that, in conjunction with schoolhouse giftedness, emphasis should also be placed on what he terms creative, productive giftedness. This type of giftedness, “describes those aspects of human activity and involvement in which a premium is placed on the development of original thought, solutions, material, and products that are purposefully designed to have an impact on one or more target audiences” (Renzulli 2016, p. 9).

In his Three Ring Conception of Giftedness, Renzulli points out that IQ is only a partial measure of giftedness, and what is equally important is an individual’s task commitment (i.e., energy brought to bear on a particular problem or specific performance area) and creativity (i.e., innovative thinking that leads to novel solutions). To a large extent, therefore, Renzulli’s work presents an alternative interpretation of the role of intelligence in giftedness.

Regrettably, in Barbados, the use of pedagogical strategies that foster the development of creative and productive giftedness is often lacking (Brissett 2022). The emphasis seems to be on schoolhouse giftedness. Many teachers express concerns that the rigidly prescribed syllabi and an examination-driven culture render it exceedingly challenging to implement innovative teaching and assessment strategies that foster the cultivation of this form of giftedness. This situation highlights the pressing need for comprehensive educational reform, which shifts the emphasis from high-stakes testing to a more process-oriented curriculum with continuous assessment, thereby granting educators greater autonomy and laying the groundwork for student-centered learning approaches that help to build on students’ intellectual ability. Fortunately, the Ministry of Education in Barbados has acknowledged the critical importance of such reform. In their draft reform document, it states that,

“With its overemphasis on academic performance, the current system does not adequately value the promotion of skills and competencies in their broadest manifestation, including those necessary to support sustainable development in the current national and global social and economic contexts. Moreover, it does not adequately prepare our students to be adults with the best chances for success as part of the community, the family, and in the workplace”.

(Ministry of Education, Technological & Vocational Training 2023, p. 31)

Despite this acknowledgment, the rate of change has been slow. As a result, frustration persists regarding what many scholars consider as a system that has outlived its effectiveness and that struggles to meet the demands and requirements of 21st-century learning. While much of what has been promised remains unfulfilled, the need for change is clear. There is a growing demand for educational stakeholders to reconsider and reimagine how education is delivered, not only to align with 21st-century learning principles but also with more modern concepts and theories of intelligence. Gardner’s work on multiple intelligences has spurred a rethinking of intelligence in schools; however, many schools still cling to traditional forms of assessment that reflect more myopic views of intelligence.

One of Gardner’s primary concerns regarding educational institutions is the almost exclusive emphasis on two forms of symbolic representation: linguistic symbolization and logical-mathematical symbolization. He contends that, although these forms of symbolic use are crucial within the academic environment, alternative forms of symbolic utilization are equally significant, both within and outside the educational context, as they exemplify the vastness of human cognitive capacities (Gardner and Hatch 1989).

Nevertheless, despite decades of research and compelling evidence supporting Gardner’s work, schools in Barbados continue to focus on the two forms of symbolic representation identified above. Students (especially at the primary level) are assessed primarily on their cognitive capacity to understand linguistic and mathematical elements. Their performance in these areas is considered the gold standard for determining the likelihood of their success, with little consideration for their other human cognitive capacities.

Indeed, educational practices in Barbados exemplify an adherence to this gold standard which prioritizes mathematics and language. Their performance in these assessment areas is interpreted as reflective of their overall intelligence, despite the possibility of exhibiting significant proficiency in other disciplines.

To address issues such as those described above, Gardner advocates for intelligence-fair assessments that reflect a more comprehensive perspective on human cognitive abilities. He contends that conventional paper-and-pencil assessments (such as the CEE) have a predisposition toward linguistic and logical evaluations. Conversely, “intelligence-fair measures seek to respect the different modes of thinking and performance that distinguish each intelligence” (p. 6).

Sternberg (2021), while acknowledging the importance of Gardner’s contributions, adopts a considerably broader perspective regarding intelligence. He presents a theory of adaptive intelligence that emphasizes the actions undertaken in our daily lives, both individually and collectively, to enhance the world around us. Sternberg contends that this approach to intelligence highlights problem-solving and innovative solutions to real-world challenges.

Adaptively intelligent people have novel and compelling ideas (creative intelligence), ensure that the ideas are logically sound and coherent (analytical intelligence), can put the ideas into practice and at some level persuade others of their usefulness (practical intelligence), and try, through their ideas, to ensure some common good (wisdom) (Sternberg 2021). This type of intelligence stands in stark contrast to general intelligence, which primarily emphasizes cognitive ability.

Unfortunately, many schools in Barbados and worldwide emphasize assessing general intelligence. That is, a student’s cognitive ability is deemed to be the most accurate measure of their intelligence. Consequently, their performance on standardized tests is seen as a dependable indicator of their intellectual aptitude. However, the problem with this approach to intelligence and testing is that it remains decontextualized from everyday personal and cultural realities that confront people in their daily lives. These include realities about survival and overcoming challenges, realities that require people to adapt. Sternberg argues that these standardized tests, while useful, do not assess the type of adaptive intelligence that is required in the real world. He identified sixteen key differences between standardized test problems (general intelligence) and adaptive intelligence problems. He posits that the emphasis on general intelligence in schools does not provide an adequate foundation for preparing students to address complex, real-world problems that require problem-solving, innovation, and creativity. Instead, he argues, the problems presented are often linear, and the answers are considered either right or wrong (Squalli and Wilson 2014; Sternberg 2021).

Sternberg argues that these major differences in focus and scope are what make it challenging for students to transfer what they learn in school to the real world. Research by Warrican et al. (2024) in Barbados raises concerns about educational practices in this area. They found that, among the sample of primary and secondary schools that participated in their study, essential skills such as problem-solving, creativity, and critical thinking were not prominently featured in the educational programs offered by schools.

### Contemporary Measures of Intelligence

Despite compelling evidence indicating that the educational system in Barbados remains predominantly anchored in traditional conceptualizations of intelligence and ability assessments, which emphasize linguistic and numerical skills, it is imperative to recognize that measurements of intelligence have undergone significant advancement. For instance, the Woodcock-Johnson IV Tests of Cognitive Abilities evaluate seven distinct cognitive faculties, namely Comprehension, Fluid Reasoning, Visual Processing, Short-term Working Memory, Auditory Processing, Cognitive Processing Speed, and Long-term Retrieval (Hajovsky et al. 2023). The Wechsler Intelligence Test also focuses on similar measurements (e.g., Fluid Reasoning, Working Memory and Processing Speed) (Nazari et al. 2024).

What these contemporary measures of intelligence highlight is the broadening of the concept of intelligence. They signify a significant shift from early measures of intelligence. Nevertheless, the Barbadian education system still seems to driven by an apparent fixation on the primary components of intelligence (Brissett 2022). This reluctance to embrace more contemporary views and measures of intelligence possibly stems from deep-seated cultural roots that emerged from the country’s colonial history.

## 6. Towards a Broader Conceptualization of Intelligence: Implications for Teaching and Learning in Barbadian Schools

As the preceding discussion suggests, substantial educational reforms are necessary for Barbados to establish an education system that aligns with current theory and research in educational psychology. This transformation needs to include a re-evaluation of the understanding of intelligence. As evidenced by recent literature (see, for example, Brissett 2022; De Lisle 2012; Hornby 2023; Warrican et al. 2024), the way intelligence is perceived and conceptualized in Barbados has profound implications for how educators instruct and assess students. Furthermore, the literature suggests that the currently used conventional teaching and assessment strategies, which are based on traditional and, to a considerable extent, narrow perspectives of intelligence, can be counterproductive and hinder the development of student potential. The work of renowned intelligence theorists, such as Gardner and Sternberg, provides a strong foundation for expanding traditional views of intelligence and incorporating more advanced approaches that consider critical learning elements, including the use of a diversity of instructional methods in the classroom.

Frameworks such as the Universal Design for Learning, through its strengths-based approach, provide a blueprint for teaching that reflects contemporary views of intelligence, while promoting more inclusive learning environments. Research by Nasri et al. (2021) supports this position. Their findings indicated that when a combination of UDL and Multiple Intelligence approaches was used, students were more engaged and excited about learning STEM subjects. They enjoyed learning in the UDL-MI-oriented STEM program. The authors note that students were surprised by the unusual learning activities, which included singing a song, participating in role-plays, debating with other students, drawing mental images, conducting hands-on STEM experiments, working in small groups, and presenting individual STEM projects.

Findings such as these point to the need for systemic change to the existing education structure in Barbados. They highlight an urgent need for officials to rethink the policy and dynamics of the education system, not only in terms of structural reforms, but also how teaching and learning are facilitated. While some educators may be willing to explore more contemporary teaching practices that consider not only multiple intelligences but also multiple ways of viewing intelligence, they may be hesitant to do so because of the examination-driven culture, where a high premium is placed on students’ success on high-stakes written examinations, such as Caribbean Secondary Education Certificate and the Common Entrance Examination.

In light of the above, the Ministry of Educational Transformation in Barbados should consider nurturing an educational culture that empowers teachers to have greater autonomy in instructional methods. Although some critics may argue that autonomy already exists, the reality is that prescriptive curricula and assessment methods often dictate teachers’ pedagogical strategies (Parcerisa et al. 2022). When the curriculum and assessments are prescriptive, it poses a greater challenge for teachers to employ innovative teaching methods that reflect contemporary views of intelligence.

### Confronting the Realities of Rethinking Intelligence and Fostering Change

While rethinking traditional views of intelligence in Barbados offers inherent benefits, the challenges that come with this shift of thought must not be overlooked. For instance, although there are calls to abolish the CEE because of its colonial history and exclusive focus only on math and language skills, research evidence (Berkowitz and Stern 2018; Breit et al. 2024; Gygi et al. 2017; Hall 2015) suggests that these proxy measures of intelligence and ability have proven valuable in predicting students’ educational success. Hall (2015), for example, found that there was a moderate relationship (r = 0.54) between student performance on the CEE and CSEC. Findings such as these highlight the predictive value of what Sternberg refers to as general intelligence tests. As such, simply removing or replacing these measurements is easier said than done.

Additionally, the pervasive examination-oriented culture in Barbados presents a significant barrier to fully embracing contemporary views of intelligence and ability testing, such as those proposed by theorists such as Sternberg and Gardner. Making this shift will mean moving away from over five decades of using the CEE as a basis for transfer from primary to secondary education, that will not be an easy task. While other countries in the Eastern Caribbean have successfully transitioned to the Caribbean Primary Exit Assessment (CPEA), which offers a more holistic assessment approach, Barbados appears to be clinging to traditional assessment practices that many argue have outlived their usefulness (De Lisle 2012; Hornby 2023; Warrican 2021). A cultural shift in practice and mindset will be required

Moreover, although IQ testing has evolved, there is still a growing concern that IQ tests and cognitive ability measures, such as the CEE, do not prepare students to solve real-world problems (Frederick 2009). This is concerning. Frederick (2009), for example, argues that IQ tests fail to measure abilities that are critical to making sound judgments in real-life situations. As we move further into the 21^st^ century and with the emergence of new technologies (e.g., artificial intelligence), other skills and character traits (e.g., teamwork, digital literacy, emotional intelligence, perseverance, drive, etc.) not currently measured by IQ will become more valued. Our capacity to adapt to these changes (Sternberg 2021) will also be critical.

Unfortunately, even the most contemporary measures of intelligence do not measure important non-cognitive factors such as grit, social skills, and emotional regulation, among other crucial elements (Duckworth 2006). Furthermore, research shows that non-cognitive factors are also good predictors of academic success. For example, Avanesian and Rozhkova (2025), found that non-cognitive skills such as open-mindedness and emotional regulation contribute significantly to academic success. However, some scholars (see, for example, Gardner 1987; Sternberg 2021) have argued that this can be achieved through implementing more authentic assessments.

Although advocating for more practical and authentic assessments that promote problem-solving, innovation, and creativity is justified as a way to accommodate learners with diverse cognitive abilities, developing such assessments requires careful planning and thorough preparation due to the inherent challenges involved in this approach. For example, based on a meta-analysis of studies on teacher ratings of students’ creativity and students’ divergent thinking, Gralewski and Karwowski (2019) concluded that teachers’ ratings should be used cautiously when assessing student creativity. Furthermore, assessment methods and pedagogical strategies such as project-based learning require intense planning and have been criticized for their high levels of subjectivity (Meng et al. 2023). Furthermore, the pressure to prepare students for high-stakes examinations may make teachers less willing to teach and assess in ways that encourage creativity (Bullard and Bahar 2023).

## 7. Conclusions

Most contemporary intelligence theorists agree that the traditional conceptualization of intelligence as a singular unitary construct presents a somewhat limited perspective of the concept. Theorists such as Sternberg, Gardner, and Renzulli have advocated for a more dynamic understanding of intelligence, emphasizing that it is not confined to academic abilities, particularly mathematical and linguistic capabilities, or performance on IQ assessments. Furthermore, IQ tests like the Wechsler Intelligence Scale, the Woodcock-Johnson IV, the Kauffman Assessment Battery for Children II, and the Multidimensional Aptitude Battery II assess at least five to seven distinct cognitive abilities (Meyer and Reynolds 2022).

Despite contemporary research and theory on intelligence, educational practices in Barbados continue to be markedly influenced by outdated perspectives and measures of this concept. An important part of this reluctance to change is the persistent challenges in transitioning away from traditional perspectives on intelligence. However, to adequately prepare our students with the necessary skills to thrive in the 21st century, it is essential to embrace a broader understanding of intelligence, one that prioritizes problem-solving, innovation, and creativity, while also recognizing and fostering diversity and inclusivity within the learning environment. At the same time, education practitioners and policymakers must remain cognizant of the practical challenges (e.g., heavy syllabus, an exam-oriented culture, low teacher morale, reluctance to move away from outdated teaching methodologies, teacher bias, etc.) of implementing such changes.

While the challenges presented above are key points of concern, learning models, such as Universal Design for Learning, and contemporary theories of intelligence, like those proposed by Sternberg and Gardner, provide valuable frameworks for navigating this process and establishing the foundation for a transformative shift in teaching practices that align with more contemporary views of intelligence.

Nevertheless, to achieve this transformation, it is imperative to change the predominant focus away from high-stakes testing and other proxy measures of intelligence, thereby granting educators greater autonomy in designing learning environments that appreciate and nurture various cognitive abilities. Furthermore, when making these shifts, it will be essential to consider the inherent strengths and limitations of both traditional and contemporary approaches to perceiving and measuring intelligence. However, if any meaningful transformation is to occur in Barbados’ education system, it will require a systemic and seismic shift away from the conventional wisdom of what constitutes intelligence.

## Data Availability

No new data were created or analyzed in this study. Data sharing is not applicable to this article.
